# High Performance of a Dominant/X-Linked Gene Panel in Patients with Neurodevelopmental Disorders

**DOI:** 10.3390/genes14030708

**Published:** 2023-03-13

**Authors:** Nino Spataro, Juan Pablo Trujillo-Quintero, Carmen Manso, Elisabeth Gabau, Nuria Capdevila, Victor Martinez-Glez, Antoni Berenguer-Llergo, Sara Reyes, Anna Brunet, Neus Baena, Miriam Guitart, Anna Ruiz

**Affiliations:** 1 Center for Genomic Medicine, Parc Taulí Hospital Universitari, Institut d’Investigació i Innovació Parc Taulí (I3PT-CERCA), Universitat Autònoma de Barcelona, 08208 Sabadell, Spain; nspataro@tauli.cat (N.S.); jptrujillo@tauli.cat (J.P.T.-Q.); cmanso@tauli.cat (C.M.); ncapdevila@tauli.cat (N.C.); vmmartinez@tauli.cat (V.M.-G.); abrunetv@tauli.cat (A.B.); nbaena@tauli.cat (N.B.); 2 Genetics Unit, Laboratory Service, Parc Taulí Hospital Universitari, Institut d’Investigació i Innovació Parc Taulí (I3PT-CERCA), Universitat Autònoma de Barcelona, 08208 Sabadell, Spain; sara.reyes@qgenomics.com (S.R.); mguitart@tauli.cat (M.G.); 3 Paediatric Unit, Parc Taulí Hospital Universitari, Institut d’Investigació i Innovació Parc Taulí (I3PT-CERCA), Universitat Autònoma de Barcelona, 08208 Sabadell, Spain; egabau@tauli.cat; 4 Rheumatology Department, Biostatistics and Bioinformatics at Institut d’Investigació i Innovació Parc Taulí (I3PT-CERCA), Universitat Autònoma de Barcelona, 08208 Sabadell, Spain; aberenguerl@tauli.cat

**Keywords:** neurodevelopmental disorders, intellectual disability, autism, gene panel, next generation sequencing, re-analysis

## Abstract

Neurodevelopmental disorders (NDDs) affect 2–5% of the population and approximately 50% of cases are due to genetic factors. Since *de novo* pathogenic variants account for the majority of cases, a gene panel including 460 dominant and X-linked genes was designed and applied to 398 patients affected by intellectual disability (ID)/global developmental delay (GDD) and/or autism (ASD). Pathogenic variants were identified in 83 different genes showing the high genetic heterogeneity of NDDs. A molecular diagnosis was established in 28.6% of patients after high-depth sequencing and stringent variant filtering. Compared to other available gene panel solutions for NDD molecular diagnosis, our panel has a higher diagnostic yield for both ID/GDD and ASD. As reported previously, a significantly higher diagnostic yield was observed: (*i*) in patients affected by ID/GDD compared to those affected only by ASD, and (*ii*) in females despite the higher proportion of males among our patients. No differences in diagnostic rates were found between patients affected by different levels of ID severity. Interestingly, patients harboring pathogenic variants presented different phenotypic features, suggesting that deep phenotypic profiling may help in predicting the presence of a pathogenic variant. Despite the high performance of our panel, whole exome-sequencing (WES) approaches may represent a more robust solution. For this reason, we propose the list of genes included in our customized gene panel and the variant filtering procedure presented here as a first-tier approach for the molecular diagnosis of NDDs in WES studies.

## 1. Introduction

Neurodevelopmental disorders (NDDs) are a group of clinically and genetically heterogeneous diseases, collectively affecting 2–5% of the general population [1]. NDDs typically manifest during childhood as a result of abnormal brain development leading to anomalies in cognitive and learning abilities, behavior, and memory. Among NDDs, intellectual disability (ID) affects 1 to 3% of the population [2] and impairs the correct functioning of conceptual skills, social abilities, and everyday self-management tasks of affected individuals [3,4], representing a significant public health problem [5]. Up to 50% of ID cases are due to genetic causes, while environmental exposure to specific teratogens, viral infections, radiation, severe head trauma and lack of oxygen to the brain are known non-genetic causes of ID and account for approximately 30% of total ID cases [6].

In the last decade, next-generation sequencing (NGS) technologies have been integrated into clinical practice and are being proposed as a first-tier approach for ID molecular diagnosis [7]. Overall, sequencing-based approaches allow the identification of the causal genetic factor in ~30% of ID cases [8,9] and facilitate the discovery of new causal genes. This fast-growing amount of information is stored in publicly accessible databases, such as SysNDD and SFARI, among others [10,11]. ID is genetically highly heterogeneous and its genetic basis is shared with other NDDs [12], explaining in part their observed comorbidity. For instance, 70% of patients affected by autistic spectrum disorder (ASD) also show ID; conversely, 40% of ID patients display ASD [13]. Several causal genes are shared among various NDDs and converge to a limited set of biological processes, such as those regulating synaptic plasticity, chromatin remodeling, gene transcription, and protein degradation [14,15]. Consequently, the analysis of the genes related to these relevant molecular pathways may allow the identification of the pathogenic genetic factors in a significant proportion of NDDs cases [12].

Although whole exome sequencing (WES) approaches allow us to interrogate the entire set of human coding regions, gene panels permit the analysis of specific genes of interest, representing a fast cost-effective solution for NDD molecular diagnosis [16]. In addition, gene panel approaches reduce the possibility of detecting variants of unknown significance and incidental findings, which may lead to uncertainty in patients rather than clarity. Many customized panels have been developed to fit with the limited bioinformatics and computational resources of most clinical diagnostic services, leading to a diagnostic rate ranging from 10 to 25%, depending on the panel used and the clinical characteristics of the patients studied [17].

In this work, we present a customized gene panel for NDD molecular diagnosis (mainly ID and ASD). Pathogenic/likely pathogenic variants were identified in 28.6% of patients. Disease severity had no effect on the diagnostic yield, which was significantly higher in patients affected by ID/global developmental delay (GDD) compared to ASD patients. Since WES approaches are replacing gene panel studies, we propose the list of 460 genes included in our custom gene panel and the filtering procedures presented here as a first-tier virtual panel approach for the fast molecular diagnosis of NDDs.

## 2. Materials and Methods

### 2.1. Patients Description and Classification

We screened 398 patients (247 males, 151 females) from the Clinical Genetics Unit at the Parc Taulí Hospital Universitari (Sabadell, Spain) between March 2019 and December 2021. Most of the selected patients were previously tested and negative for array-CGH and *FMR1* CGG expansion studies. Patients having consanguineous parents were excluded from this study. Informed consent was obtained from patients, parents or enrolled participant’s legal representatives.

Clinical characteristics were manually extracted from the patient’s electronic health record and codified using Human Phenotype Ontology (HPO) codes [18]. The list of considered patients, their demographic information, and clinical characteristics are available in Table S1.

Since all enrolled patients had a clinical diagnosis of ID/GDD or ASD, HPO codes were leveraged to classify patients into two possible subsets. The autistic spectrum disorder (ASD) subset comprised 76 patients with HPOs related to autistic behavior (HP:0000729, HP:0000735, HP:0000728, HP:0008763, HP:0000758, HP:0000717, HP:0031433, HP:0000723, HP:0000753) but without HPOs related to ID/GDD (HP:0001249, HP:0001256, HP:0010864, HP:0006887, HP:0006889, HP:0002187, HP:0002342, HP:0001263, HP:0012758, HP:0100543). All the remaining 322 patients were included in the ID/GDD subset.

To test whether clinical and demographic features could predict the absence/presence of a pathogenic variant, each single HPO term used was classified into 16 possible categories representing different affected systems or clinical entities. The following categories were taken into account as predictors: “Abnormality of metabolism/homeostasis”, “Abnormality of prenatal development or birth”, “Abnormality of the cardiovascular system”, “Abnormality of the digestive system”, “Abnormality of the ear”, “Abnormality of the endocrine system”, “Abnormality of the eye”, “Abnormality of the genitourinary system”, “Abnormality of the head”, “Abnormality of the integument”, “Abnormality of the musculoskeletal system”, “Abnormality of the respiratory system”, “Abnormal nervous system morphology”, “Abnormal nervous system physiology”, “Behavioral abnormality”, “Intellectual disability”. The list of HPOs included in each category is available in Table S2. In addition, the patient’s sex and the total number of affected categories were also used as predictors in our analysis. For each considered predictor, a correlation-like measure was computed to test its association with the presence of the pathogenic variants. In particular, the Glass rank biserial correlation coefficient was computed for the predictor total number of affected categories, while ϕ correlation coefficients were computed for the rest of the considered predictors.

A random forest (RF) analysis was carried out using the R library “randomforest” to evaluate the combined predictive ability of all the considered predictors. To assess the accuracy of the model, out-of-bag (OOB) estimates were used. The model was grown using 5000 trees, which were sufficient to achieve stable OOB estimates. Data were stratified by outcome; an under-sampling without replacement technique to balance the dataset was used by randomly selecting 80% of the least frequent category (presence of a pathogenic variant). Default values were used for all the other RF parameters.

For exploratory purposes, we aimed to identify shared phenotypic profiles among patients harboring pathogenic variants (analysis of prototypes). To this end, patient’s pairwise proximities (PROX) derived from the RF classifier were transformed to dissimilarities (1-PROX) and a hierarchical clustering was performed using the Ward agglomerative method. The resulting dendrogram was used to manually identify groups of neighbor patients harboring pathogenic variants. Finally, the phenotypic features of the patients in the identified groups were compared to those of the rest of the patients.

### 2.2. Gene Panel Content

As most identified NDD genes follow a dominant inheritance model [19], a customized gene panel (NNDTauliPanel) was designed to cover the coding region of 460 autosomal dominant or X-linked genes associated with ID/GDD or ASD. Genes were extracted from the SysID database (https://sysndd.dbmr.unibe.ch/ accessed on 20 January 2019), a manually curated database of known ID-associated genes [11]. ASD-associated genes were extracted from SFARI (https://gene.sfari.org/ accessed on 20 January 2019) (score 1 or 2 genes), an evolving database for the autism research community [10]. The initial list of genes was manually reduced to fit with the library and sequencing requirements of an Illumina MiSeq platform, considering the genes ascertained to be strongly associated with ID/GDD or ASD. The Design Studio software (Illumina, Inc., San Diego, CA, USA) was used to design an enrichment library to sequence the coding regions of the final list of 460 genes. Overall, the designed probes covered a total of 1.73 Mb of coding sequences (and 20 bp intronic sequences at each side), representing 1.57% of the whole human exome and 0.05% of the whole human genome. The list of genes, exon coordinates, and obtained coverage values is available in Table S3.

### 2.3. Sequencing and Data Processing

Sequencing was performed using the NDDTauliPanel and an Illumina MiSeq platform (Illumina, San Diego, CA, USA), producing 2 × 150 nt paired-end reads. Raw data quality was assessed using FastQC software (v0.11.8) [20]; raw reads were mapped to the human reference genome (hg19) using Burrows–Wheeler aligner (BWA, v0.7.17-r1188) [21] and subsequently processed using the Genome Analysis Toolkit (GATK) [22] to remove PCR duplicates and perform base quality score recalibration. Only bases with a Phred quality score >18 were considered for variant calling and only variants with Phred-scaled confidence >10 were called using the Haplotype Caller tool from GATK (v4.0.11.0) [23]. Variant annotation was performed using WGSA pipeline (version 0.85) [24].

Coverage analysis was performed for each considered expanded exon (20 bp at each side). For every patient, an average coverage of 244× was obtained and on average 98.05 and 92.41% of the considered positions had coverage ≥ 10 and ≥50, respectively (Table S3). For *SYNGAP1*, *TUBB*, and *EHMT1*, the sequencing was not possible in 7, 3, and 1 of their exons, respectively, and genetic variation could not be interrogated in these regions (Table S3).

### 2.4. Variant Filtering

A stringent in-house filtering pipeline was developed to detect highly penetrant ultra-rare variants. Firstly, variants with a read depth < 10 and variants with an allele count > 4 in gnomAD [25] were discarded. Variants with a proportion of the alternate allele < 30% were also discarded.

Among variants passing the above filtering criteria, only those predicted to affect the protein sequence were considered for further analysis. Only loss of function (LoF) variants (stop gain, frameshift, canonical splicing, start loss), stop loss, non-frameshift, missense, and variants predicted to affect splicing were considered. Variants detected with a score ≥ 0.5 by either SQUIRLS [26], spliceAI [27], splice ADA, or splicing RF [28] were considered as variants predicted to affect splicing and thus retained.

Finally, variants were prioritized, considering as candidate those that (*i*) are reported as pathogenic/likely pathogenic in ClinVar, (*ii*) are absent in gnomAD, and (*iii*) are predicted to strongly affect protein function (LoF, non-frameshift with a PROVEAN [29] score ≤ −2.5, missense predicted to be deleterious by ≥50% of available bioinformatics predictors, variants predicted to affect splicing). For missense variants, the following bioinformatics predictors were used to assess their deleterious effect: (*i*) predictors based on functional prediction (SIFT, SIFT4G, Polyphen2-HDIV, Polyphen2-HVAR, LRT, MutationTaster2, MutationAssessor, FATHMM, MetaSVM, MetaLR, CADD, VEST4, PROVEAN, FATHMM-MKL, FATHMM-XF, fitCons, DANN, GenoCanyon, Eigen, Eigen-PC, M-CAP, REVEL, MutPred, MVP, MPC, PrimateAI, DEOGEN2), and (*ii*) predictors based on conservation scores (bStatistic, phyloP100way_vertebrate, phyloP30way_mammal, phyloP17way_primate, phastCons100way_vertebrate, phastCons30way_mammal, phastCons17way_primate, GERP++ and SiPhy).

### 2.5. Re-Analysis Variant Filtering

Since the filtering pipeline was extensively changed from its initial version, once all samples had been processed, the whole dataset was re-analyzed in January 2022, considering the most updated version of the pipeline. In addition, a different setting for the variant filtering process was implemented to also detect variants in mosaic state and variants with a low proportion of the alternate allele. Again, variants with a read depth < 10 and variants with an allele count > 4 in gnomAD were discarded. Variants with a read depth ≥ 10 and <50 were discarded when the proportion of the alternate allele was <20% for LoF variants or <25% for non-LoF variants, respectively. To detect mosaic variants, those with a read depth ≥ 50 and a proportion of the alternate allele ≥ 10% were retained. To mitigate the possible increase in false positives, a mechanism to easily identify sequencing errors was implemented. In particular, variants absent in the general population but present in >3 patients of our cohort were highlighted as potential false positive calls.

### 2.6. Copy Number Variants Detection and Filtering

Copy number variants (CNVs) were called using XHMM [30] and ExomeDepth [31]. To reduce the number of false positive CNV calls, those reciprocally sharing 80% of their length with >3 other CNVs calls were discarded. Similarly, CNVs reciprocally sharing 90% of their size with those having an allele count > 10 or an allele frequency > 0.0001 in either DGV [32] or gnomAD were discarded. Finally, CNVs called by only one software and with a Phred score < 20 for XHMM or <3 for ExomeDepth were discarded. CNVs were prioritized by considering as candidates those called with a Phred score ≥ 30 by XHMM and a Phred score ≥ 20 by ExomeDepth.

### 2.7. Exome Sequencing

In order to compare the NDDTauliPanel performance with WES approaches, for 85 out of the 284 patients with negative NDDTauliPanel, WES was performed using the KAPAHyper Exome kit (Roche) on a NovaSeq 6000 platform (Illumina) producing 2 × 50 nt paired-end reads at CNAG-CRG (Barcelona, Spain). Data processing, variant calling and filtering procedures described in Section 2.3, Section 2.4, Section 2.5 and Section 2.6 were also applied to WES data. Considering the current information stored at SysNDD and SFARI (accessed on 20 December 2022), an updated virtual version of the NDDTauliPanel was generated. To filter variants located in genes following a recessive inheritance model, compound heterozygous and homozygous variants with a frequency ≥ 0.1% or found in homozygosity in >4 individuals in gnomAD were discarded. The filtering criteria described in Section 2.4, Section 2.5 and Section 2.6 were applied for dominant and X-linked genes.

### 2.8. Variant Validation and Classification

A manual search was carried out for the variants passing the filtering criteria to check if the clinical characteristics described in the literature were consistent with those of the patient carrying a given variant of interest. The inheritance pattern of the selected variants was assessed using Sanger sequencing for SNPs and indels, while MLPA or array-CGH was used for putative CNVs. Variants were classified following the American College of Medical Genetics and Genomics and the Association for Molecular Pathology (ACMG/AMP) guidelines [33] and the recommendations provided by the Sequence Variant Interpretation working group at ClinGen for criteria PVS1, PM2 and PS2/PM6 (https://clinicalgenome.org/working-groups/sequence-variant-interpretation/ accessed on 4 October 2022). Variants classified as pathogenic and likely pathogenic were submitted to ClinVar.

## 3. Results

### 3.1. Diagnostic Yield, Variant Types, and Genes

The NDDTauliPanel was used for the molecular diagnosis of 398 patients with an average age of 14.5 years old (standard deviation = 11.20 years old) affected by either ID/GDD or ASD. On average, only six variants per sample were kept after filtering, and of those, only 1.6 variants per sample were prioritized as candidates. Overall, 99.73% of the detected variants were eliminated during the filtering process.

According to the ACMG/AMP criteria and ClinGen recommendations, 80 pathogenic and 35 likely pathogenic variants in 114 patients were identified (one patient carried two pathogenic variants), leading to a final 28.6% diagnostic yield (Table S4).

Among these pathogenic/likely pathogenic variants, 73.9% were *de novo*, 10.4% were inherited, 8.7% were not inherited by the single available parent, and for 7% of variants inheritance could not be assessed (Table 1). LoF variants represented 53.9% (62 variants) of those reported as pathogenic/likely pathogenic, while 35.7% (41) and 4.3% (5) variants were missense and non-frameshift, respectively. In addition, CNV calling using the NGS data allowed the identification of three pathogenic deletions and two likely pathogenic duplications. Finally, two variants predicted to affect the splicing process were identified, a pathogenic *de novo* intronic variant in *SETD5* and a pathogenic *de novo* synonymous variant in *ARID1B* (Table 1, Table S4). In both cases, mRNA analysis had been previously reported in the literature, demonstrating their effect on splicing and the generation of a truncated protein product [34,35].

Pathogenic variants were identified in 83 different genes. For 25 different genes, pathogenic/likely pathogenic variants were detected in more than one patient (Table S4). Among those, five *de novo* pathogenic variants were identified in *ANKRD11,* causing KBG syndrome, characterized by macrodontia of the upper central incisors, characteristic facial features, short stature, developmental delay/intellectual disability, and behavioral issues [36].

Remarkably, a *de novo* missense and a *de novo* frameshift deletion were detected in two male patients in the *FMR1* gene. The patient carrying the frameshift deletion showed clinical characteristics compatible with Fragile X syndrome. Instead, the patient harboring the missense variant was affected by severe ID and presented clinical features not suggesting Fragile X syndrome. Pathogenic variants in the coding region of *FMR1* are causal in <1% of Fragile X syndrome cases and only a few dozen patients with point variants have been reported in the literature to date [37].

A *de novo* likely pathogenic duplication predicted to affect exons 2–14 was initially identified from NGS data in *WDR26* and confirmed by array CGH to involve the whole sequence of *WDR26*. To our knowledge, no patients have been reported in the literature with duplications involving *WDR26*. In addition, a splicing likely pathogenic variant was identified in *WDR26* for a second patient and a *de novo* recurrent missense likely pathogenic variant was also detected in *FBXO28* for a third patient. *WDR26* and *FBXO28* are located within the 1q41-q42 deletion syndrome critical region, a rare cause of intellectual disability, seizures, facial dysmorphia, and multiple anomalies [38]. All three patients with variants in *WDR26/FBXO28* were affected by severe ID, epilepsy, dysmorphia, behavioral abnormalities, and central nervous system alterations, including incomplete hippocampal inversion, corpus callosum hypoplasia, or cerebellar atrophy. None of our patients showed the characteristic multi-system features of patients affected by 1q41-q42 deletion syndrome [39].

### 3.2. Data Re-Analysis

Once all samples had been processed, the whole dataset was re-analyzed to capture putative variants in mosaic state and variants with a low proportion of the alternate allele. Comparing original and re-analysis data, 93 variants previously not detected were identified and 15 were selected for further analysis.

Of those, a frameshift insertion in *OTUD7A* and 5 CNVs resulted in being false positives. Finally, five variants (two missense, one frameshift, one stop gain, one CNV deletion) were classified as pathogenic and one missense variant as likely pathogenic (Table S5), increasing the diagnostic yield from an initial 27.1% to the final 28.6%. Among the six pathogenic/likely pathogenic variants detected in the re-analysis, two were identified reducing the original alternate allele frequency threshold from 30% to 25%; three variants were identified using the filtering criteria designed to detect variants in the mosaic state (Table S5). However, when validated through Sanger sequencing, those variants appeared to be in heterozygous state (Figure S1).

Remarkably, among the eight SNV and indels considered in the re-analysis (Table S5), only a frameshift insertion in *OTUD7A* resulted to be false positive. Although our results are based on a limited number of observations, our findings suggest that for these types of variants the joined usage of relaxed quality criteria and a mechanism to easily detect sequencing error could significantly increase the sensitivity, with a minor effect on the number of false positives generated.

### 3.3. Sex and Patient Subset Analysis

Clinical characteristics were manually retrieved from electronic health records and codified using HPO terms (Table S1). Taking advantage of the codified clinical features, patients were classified either in ID/GDD or ASD subsets, as explained in Materials and Methods (Section 2.1). In total, 322 patients (180 males, 142 females) were included in the ID/GDD subset, and 76 patients (67 males, 9 females) in the ASD subset.

The diagnostic rate ranged from 33.2% for the ID/GDD subset to 9.2% for the ASD subset (*p*-value = 5.69 × 10^−5^ for Χ^2^ test), suggesting that the designed custom gene panel has a very high performance for patients affected by ID/GDD. Although the diagnostic yield was much lower in ASD patients, a recent meta-analysis evaluating several gene panels demonstrated that the diagnostic rate in ASD patients ranges from 0.22 to 10% [40] indicating that NDDTauliPanel has a high performance for ASD patients as well.

Males were significantly enriched in the whole cohort (*p*-value = 1.494 × 10^−6^ for Χ^2^ test) and even more in the ASD subset (*p*-value = 4.648 × 10^−11^ for Χ^2^ test). However, pathogenic/likely pathogenic variants were identified in a higher proportion of females (*p*-value = 0.03 for Χ^2^ test) (Table 1). As reported by previous studies, our finding supports a female protecting (or male susceptibility) effect, in which females require a higher mutational burden to develop NDD clinical manifestations [41,42,43]. Pathogenic variants in the 106 X-linked genes included in the NDDTauliPanel were detected in 7.9 and 2.8% and accounted for 20.4 and 11.4% of total pathogenic variants in females and males, respectively. These findings indicate that X-linked genes do not account for the higher number of affected males among NDD patients (Table S4). Most variants in X-linked genes in females were *de novo* (8 out 10 tested) and four of them were located in genes known to escape X-inactivation *(DDX3X*, *USP9X* and *IQSEC2*), for which pathogenic variants are usually lethal in males and clinical manifestations are only observed in females. All the other variants identified in X-linked genes were located in genes not known to escape X-inactivation and for which females often show a milder phenotype than males.

### 3.4. Clinical Profiling

For 261 patients in the ID/GDD subset, the severity of the ID was extracted from the patient’s electronic health record (Table S1). In detail, 60 borderline, 111 mild, 55 moderate, and 35 severe ID patients were identified. No significant differences in the diagnostic rate were observed among ID categories (*p*-value = 0.21 for Χ^2^ test), even though the diagnostic yield ranged from 28.3% in borderline patients to 37.1% in patients with severe ID (Table S1).

**Figure 1:**
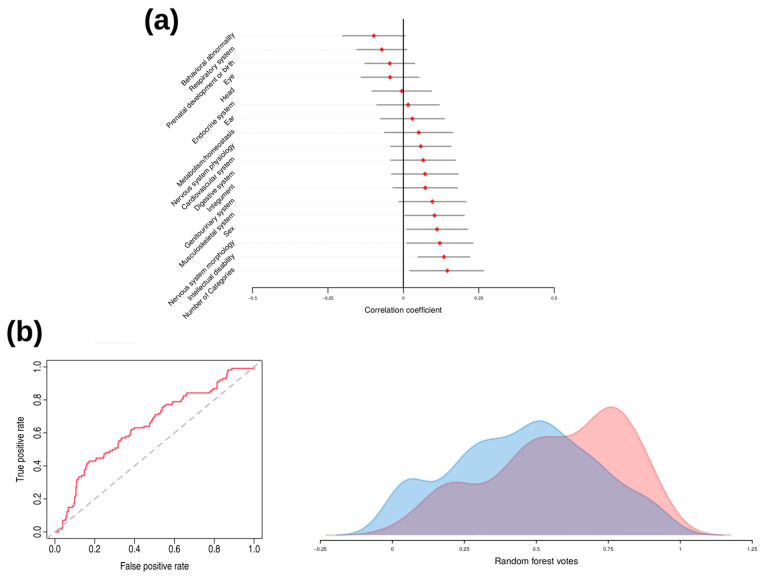
Association of phenotypic variables with the presence of pathogenic variants and random forest analysis results. (**a**) Correlation coefficients and corresponding 95% confidence intervals for the phenotypic variables described in Section 2.1. The Glass rank biserial correlation coefficient was obtained for the variable “Number of categories”, while for all the remaining variables, the ϕ correlation coefficient was computed. (**b**) Receiver operating curve (ROC) and area under the curve (AUC) for the prediction model in the random forest analysis (left). On the right are represented the distributions of random forest votes between patients with identified pathogenic variant (red) and the rest (blue).

In order to check if phenotypic features could predict the presence of a pathogenic variant, correlation coefficients and their confidence intervals were computed for the 16 HPO categories, the number of affected HPO categories, and the sex. Although each single variable had a poor predictive power, the predictors: number of affected HPO categories, sex, ID, abnormalities in the nervous system morphology, and abnormalities of the musculoskeletal system positively associated with the presence of pathogenic variants (Figure 1a).

Subsequently, a random forest analysis was performed to assess the joint predictive power of the considered variables in predicting the presence of a pathogenic variant. Overall, the model showed a sensitivity of 64%, a specificity of 59.6%, an accuracy of 60.1% and area under the curve (AUC) of 0.63 (CI 0.57–0.69) (Figure 1b, left), suggesting that the set of variables poorly predicts the presence of pathogenic variants. Consistently with this weak discrimination ability, patients with and without pathogenic variants showed a small although significant difference in the probabilities provided by the random forest model (median differences = 0.12, Mann–Whitney U-test *p*-value = 6.56 × 10^−6^) (Figure 1b, right). Although the considered variables have poor predictive power, this evidence suggests that some phenotypic differences exist between patients with identified pathogenic variant and the rest.

To test whether patients could be grouped together according to their shared phenotypic features, an exploratory analysis was conducted on the proximities derived from the random forest model (prototype analysis). Three different groups enriched with patients harboring pathogenic variants were identified (Figure S2). A diagnostic yield of 56, 46.3, and 50% was achieved for the 25, 54, and 26 patients in groups 1, 2, and 3, respectively (Table S6). The phenotypic features defining the clinical profiles of these three groups are available in Table S6.

### 3.5. WES Analysis

For 85 out of the 284 patients with a negative NDDTauliPanel, WES was performed and pathogenic/likely pathogenic variants were identified in 16 patients (Table S7). Of those, variants in compound heterozygous or homozygous state were detected in five patients in recessive genes not covered by the NDDTauliPanel. For ten patients, pathogenic/likely pathogenic variants were located in genes related to dominant forms of ID but not included in our design (two variants in *AP1G1*, one variant in *JARID2*, *RHEB*, *TAF1*, *TRPM3*, *SPEN*, *MAP1B*, *SHANK1*, and *GRIA2*). Finally, for a pathogenic variant located at the 5′ end of exon 24 of *SHANK3*, the coverage obtained from the NDDTauliPanel did not reach the minimal requirements for considering the variant. In contrast, the 46x coverage obtained from WES was sufficiently high to allow its detection.

## 4. Discussion

The advent of high-throughput sequencing technologies has revolutionized the molecular diagnosis of neurodevelopmental disorders (NDDs) and has facilitated the identification of a growing number of associated genes and the detection of shared molecular pathways playing a central role in their etiopathogenesis. Although current technologies allow us to interrogate the whole genome, generally, molecular diagnosis is obtained considering a portion of the genome, either the exome or a limited list of genes related to a disease of interest. Using an in-house targeted gene panel sequencing strategy, a 28.6% global diagnostic yield was obtained in 398 patients affected by ID/GDD and ASD.

A higher diagnostic yield was observed in females, despite the higher proportion of males in the cohort, supporting a female protecting effect. As previously reported, the diagnostic rate was significantly higher in patients affected by ID/GDD than those solely affected by ASD [44]. Interestingly, most of the NDD patients were affected by borderline/mild forms of ID, suggesting that NDDTauliPanel represents a powerful solution independent of the patient’s ID level. Although patients included in this study were recruited only by one hospital, which could lead to a possible ascertainment bias and a higher diagnostic yield, no differences in the diagnostic rate were observed between the ID severity levels, as previously reported for pathogenic CNVs in patients with ID and comorbid psychiatric disorders [45].

Our NDDTauliPanel outperforms most of the gene panel approaches for both ID/GDD [9] and ASD patients [40]. In particular, a higher diagnostic rate was achieved using the NDDTauliPanel compared to 22 out of the 26 gene panel approaches having a sample size >100 described in a recent meta-analysis [9] (Figure S3). The remaining four studies were focused on early onset epilepsy [46,47], and early infantile onset developmental and epileptic encephalopathies [48,49], for which a much higher diagnostic yield was reported in the literature compared to ID/GDD and ASD [50].

Despite the high diagnostic rate obtained from the NDDTauliPanel, our results suggest that WES solutions may represent a more powerful approach, allowing the detection of pathogenic variants in genes with recessive inheritance and those recently described to be associated with NDDs. The NDDTauliPanel was designed using the information available in January 2019 and represents only a subset of all the NDD genes discovered to date. Considering 480 exomes previously analyzed by our laboratory, an average of 115x coverage was obtained for the genomic positions of the pathogenic/likely pathogenic variants detected using the NDDTauliPanel, demonstrating that current WES approaches guarantee high coverage over all variants identified in this work. However, in WES studies, identifying the causal genetic factors is more time-consuming due to the higher number of variants generated. Considering our WES data and the current size of SysNDD and SFARI, 27 times more variants were obtained in WES compared to NDDTauliPanel after variant filtering, which significantly increases the cost and the turnaround time compared to the gene panel setting.

Leveraging the codified phenotypic features retrieved from the patients’ electronic health records, we aimed to identify the clinical profile of patients with identified pathogenic variants. As a whole, the considered phenotypic features poorly predicted the presence of a pathogenic variant. However, our results demonstrate that specific subgroups of patients show particular phenotypic features. Although the identified clinical profiles cannot be introduced into current clinical practice, we believe that the use of codified clinical features joined with deep-phenotyping techniques may improve decision making for genetic testing.

Considering the constant drop in sequencing price, the computational costs and all the evidence provided by this work, we propose the list of 460 genes included in our custom gene panel and the variant filtering procedure presented here as a first-tier approach for the molecular diagnosis of NDDs in WES studies. The joint usage of (*i*) the proposed virtual gene panel, (*ii*) a very stringent filtering procedure, (*iii*) procedures for the identification of sequencing errors, (*iv*) multiple softwares for the detection of CNVs and cryptic splicing variants, and (*v*) criteria for variant prioritization may result in a limited list of candidate variants, leading to the identification of the pathogenic genetic variants in approximately 30% of NDD patients in WES studies.

## 5. Conclusions

Nowadays, both WES and whole genome sequencing (WGS) are cost-effective, although at present for WGS settings, the computational resources available in most clinical laboratories are not sufficient for the analysis of hundreds of patients. For this reason, currently WES studies represent the most common solution adopted by clinical laboratories for the molecular diagnosis of genetic diseases. The major challenge in analyzing both WES and WGS data is represented by the vast number of variants generated and their subsequent interpretation. Although WES solutions are replacing gene panel studies, the results presented here highlight the importance of stringent filtering criteria for the efficient detection of causal genetic variants. To this end, we propose the implemented filtering procedure and the list of 460 genes used in the NDDTauliPanel as a first-tier virtual panel for WES studies in patients affected by dominant or X-linked forms of NDDs.

## Figures and Tables

**Table 1:** Diagnostic yields, inheritance patterns and variant types for pathogenic/likely pathogenic variants.

		ALL	ID/GDD	ASD
	Patients (M/F)	398 (247/151)	322 (180/142)	76 (67/9)
**Diagnostic yield**	All	28.6%	33.2%	9.2%
	M	24.7%	30.0%	10.4%
	F	35.1%	37.3%	0.0%
**Inheritance Pattern**	*de novo*	73.9% (85)	64.8% (79)	85.7% (6)
	Inherited	10.4% (12)	10.2% (11)	0.9% (1)
	X-linked (M/F)	16.5% (7/12)	17.6% (7/12)	0.0% (0)
	Uncertain/NA	15.7% (10/8)	16.7% (10/8)	0.0% (0/0)
**Variant type**	LoF	53.9% (62)	51.8% (56)	85.7% (6)
	Missense/in frame	40.0% (41/5)	41.7% (41/4)	14.3% (0/1)
	Cryptic splicing	1.8% (2)	1.9% (2)	0.0% (0)
	CNVs (DEL/DUP)	4.3% (3/2)	4.6% (3/2)	0.0% (0/0)

ID/GDD and ASD subsets explained in Section 2.1; the total numbers of patients or variants are reported in parentheses; M = males; F = females; NA = not available; DEL = CNV deletion; DUP = CNV duplication.

## Data Availability

The raw data are not publicly available due to privacy and ethical restrictions. The data related to the results presented in this study are available in the Supplementary Material.

## Supplementary Materials

The following supporting information can be downloaded at: https://www.mdpi.com/article/10.3390/genes14030708/s1, Table S1: Patients demographic and clinical description; Table S2: HPO categories; Table S3: Coverage analysis; Table S4: Identified pathogenic and likely pathogenic variants; Table S5: Re-analysis candidate variants; Table S6: Phenotypic features of groups of patients obtained from the supervised hierarchical grouping algorithm applied on proximities derived from random forest analysis; Table S7: Identified pathogenic and likely pathogenic variants in WES; Figure S1: Inheritance pattern for variants in putative mosaic state; Figure S2: Dendrogram applied to the dissimilarities derived from the random forest classifier; Figure S3: Diagnostic rate comparison between NDDTauliPanel and the gene panel studies having sample size > 100 in Stefinsky et al., 2021.
