# Supplementary material for: High Performance of a Dominant/X-Linked Gene Panel in Patients with Neurodevelopmental Disorders
Source: Genes (Basel). 2023 Mar 13;14(3):708. doi: 10.3390/genes14030708 (PMC10048137; doi:10.3390/genes14030708)
Supplement: Supplementary file 1 [file genes-14-00708-s001.zip › SupplementaryMaterial.pdf]

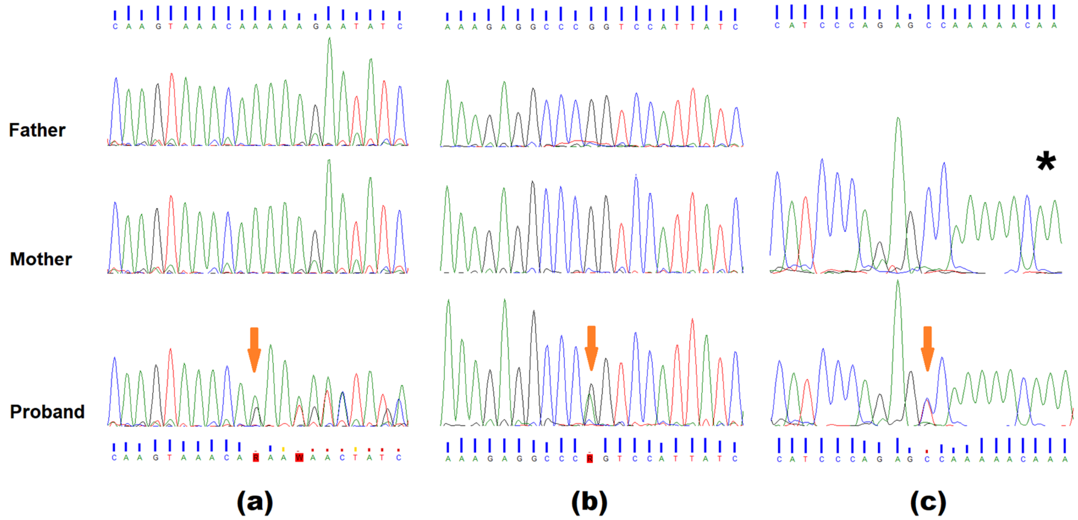

Figure S1. Inheritance pattern for variants in putative mosaic state. Orange arrows indicate the identified single point causal variant. (**a**) Inheritance pattern for *ASXL3* NM_030632.3:c.4890_4893del. (**b**) Inheritance pattern for *TLK2* NM_006852.6:c.1637G>A. (**c**) Sanger sequencing for *PHIP* NM_017934.7:c.894G>A in proband and a non-related control indicated by (*).

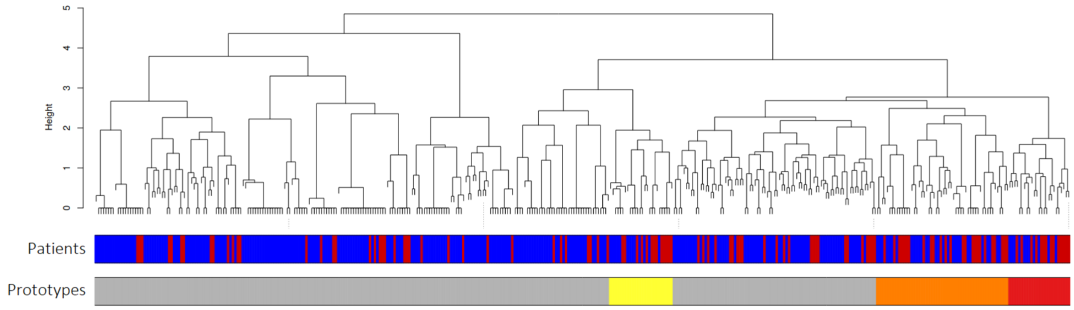

Figure S2. Dendrogram applied to the dissimilarities derived from the Random Forest classifier. The "Patients" bar represents patients with identified pathogenic variant (red) and patients for which a pathogenic variant was not identified (blue). The yellow, orange and red blocks in the bar "Prototypes" highlight the manually identified groups of patients enriched of patients with identified pathogenic variants.

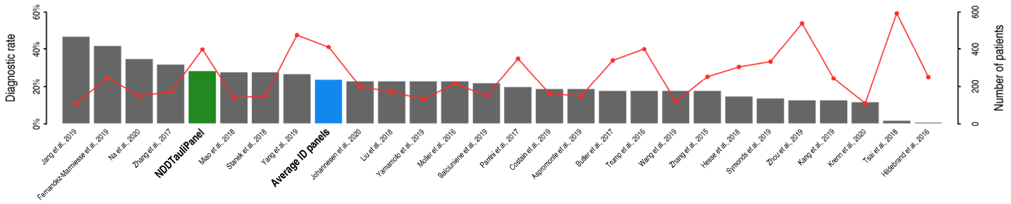

Figure S3. Diagnostic rate comparison between NDDTauliPanel and the gene panel studies having sample size >100 in Stefinsky et al., 2021. The bars represent the diagnostic rate represented in the y-axis; red dots and line represent the gene panel sample size represented in the z-axis. Green and blue bars represent the diagnostic rate of the NDDTauliPanel and the average diagnostic rate of the rest of genel panel studies considered, respectively.
